# Transcriptomic analysis of the antimicrobial activity of prodigiosin against *Cutibacterium acnes*

**DOI:** 10.1038/s41598-023-44612-7

**Published:** 2023-10-13

**Authors:** Hyun Ju Kim, Moo-Seung Lee, Se Kyoo Jeong, Sang Jun Lee

**Affiliations:** 1 Department of Systems Biotechnology, and Institute of Microbiomics, Chung-Ang University, Anseong, 17546 Republic of Korea; 2 Environmental Diseases Research Center, Korea Research Institute of Bioscience and Biotechnology (KRIBB), Daejeon, 34141 Republic of Korea; 3 Department of Biomolecular Science, KRIBB School of Bioscience, Korea University of Science and Technology (UST), Daejeon, 34113 Republic of Korea; 4 Research Division, Incospharm Corp., Daejeon, 34036 Republic of Korea

**Keywords:** Antimicrobials, Bacteria, Microbiology

## Abstract

Prodigiosin, a red pigment produced by *Hahella chejuensis*, a marine-derived microorganism, has several biological functions, including antimicrobial activity and inflammatory relief. In this study, the antibacterial activity of prodigiosin against skin microorganisms was explored. Paper disc assay on skin bacterial cells revealed that *Cutibacterium acnes* related to acne vulgaris highly susceptible to prodigiosin. MIC (Minimal Inhibitory Concentration) and MBC (Minimal Bactericidal Concentration) were determined on *Cutibacterium* species. The RNA-seq analysis of prodigiosin-treated *C. acnes* cells was performed to understand the antibacterial mechanism of prodigiosin. Among changes in the expression of hundreds of genes, the expression of a stress-responsive sigma factor encoded by *sigB* increased. Conversely, the gene expression of cell wall biosynthesis and energy metabolism was inhibited by prodigiosin. Specifically, the expression of genes related to the metabolism of porphyrin, a pro-inflammatory metabolite, was significantly reduced. Therefore, prodigiosin could be used to control *C. acnes*. Our study provided new insights into the antimicrobial mechanism of prodigiosin against *C. acnes* strains.

## Introduction

Prodigiosin is a natural red pigment first isolated from *Serratia marcescens*. It is produced by various microorganisms, including strains of *Streptomyces coelicolor*, *Vibrio* spp., and *Hahella chejuensis*^1–3^. Its chemical structure is composed of 2-methyl-3-*n*-amyl-pyrrole and 4-methoxy-2,2ʹ-bipyrrole-5-carbaldehyde. The biosynthesis pathway of prodigiosin is well known from the *pig* gene cluster in *S. marcescens*. When 2-methyl-3-*n*-amyl-pyrrole and 4-methoxy-2,2ʹ-bipyrrole-5-carbaldehyde are synthesized in cells, the PigC enzyme promotes the condensation of both pyrroles to produce prodigiosin^4^.

The physiological role of prodigiosin in bacteria has not yet been clearly defined. However, studies have shown that prodigiosin is a typical secondary metabolite that appears in later bacterial growth stages. It has a trypanolytic activity in *S. marcescens* strains or is a product of an overflow in primary metabolism^5^.

Prodigiosin has several biological activities. First, it has antibacterial activities against bacteria such as *Bacillus subtilis*, *Staphylococcus aureus*, and *Pseudomonas aeruginosa*^6–8^. It also exhibits an antibacterial activity against red tide-causing bacteria^9,10^. In addition, it has an antiprotozoal activity against protozoa causing malaria and Chagas disease^11^. It also has anti-inflammatory and antitumor activities^12,13^. Therefore, it has high applicability as a pharmaceutical, but further studies on its pharmacodynamics and toxicity should be performed for its drug application^3^.

Prodigiosin may be used as a microbial pigment by achieving a high yield through microbial strain improvement; particularly, prodigiosin can be used as a functional cosmetic pigment material because it is red. It is effective against ROS (Reactive Oxygen Species) generation, inflammation, and cytotoxicity following UV (Ultra Violet) exposure in skin keratinocytes^12^.

In human skin, various microorganisms, including *Staphylococcus*, *Corynebacterium*, and *Cutibacterium* form a microbial community^14^. *Cutibacterium acnes* strains help maintain skin health by inhibiting pathogen invasion, but they are also associated with acne, a chronic inflammatory disease in adolescents and young adults^15–17^. Therefore, *Cutibacterium* species are considered commensal or opportunistic pathogens^18^.

In this study, the antibacterial effect of prodigiosin produced from marine-isolated *H. chejuensis* on skin microorganisms was tested. *C. acnes* strain, an opportunistic pathogen, was then treated with prodigiosin, and the antibacterial mechanism of prodigiosin was studied through transcriptomic analysis. Thus, this study provided a basic understanding of the antibacterial mechanism of prodigiosin and its applicability as a functional cosmetic ingredient.

## Results

### Antimicrobial activity against skin commensal microbes

**Figure 1:**
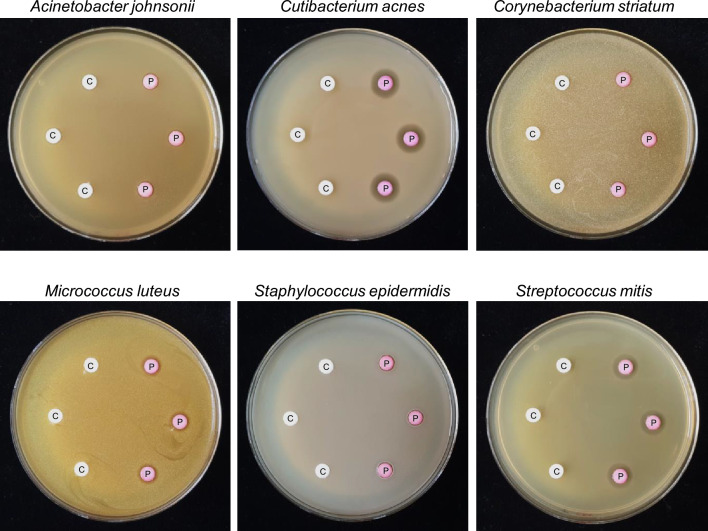
Paper-disc assay of the antibacterial activity of prodigiosin against six skin commensal microbe strains. C and P indicate the negative control (ethanol only) and prodigiosin (10 μg) treatment, respectively.

A paper-disc assay was performed to confirm the antibacterial activity of prodigiosin against six skin commensal microbe strains (*A. johnsonii*, *C. acnes*, *C. striatum*, *M, luteus*, *S. epidermidis*, and *S. mitis*). In all six strains, no inhibitory zone was observed in an ethanol-absorbed paper disc as the control. In *A. johnsonii*, *C. striatum*, and *S. epidermidis* strains, no inhibitory zone was observed in the prodigiosin (10 μg)-absorbed paper disc. In *C. acnes*, *M. luteus*, and *S. mitis*, approximately 3.1, 1.1, and 2.2 mm of inhibitory zone were observed in the prodigiosin (10 μg)-absorbed paper disc (Fig. 1). Prodigiosin showed the largest inhibition zone against *C. acnes* strain among the six tested microbe strains. In the *C. acnes* strain, the size of the inhibition zone increased as the amount of the prodigiosin-absorbed paper disc (Figure S1) increased. *C. avidum* (KCTC 5339) and *C. granulosum* (KCTC 5747) strains from *Cutibacterium* also had an inhibition zone by prodigiosin.

The MIC and MBC tests were performed to quantify the antibacterial activity of prodigiosin. *A. johnsonii* strain showed a bacteriostatic effect with MIC, MBC, and MBC/MIC ratio of 3.125 μg/ml, 25 μg/ml, and 8, respectively. The other strains showed a bactericidal effect with MBC/MIC ratio of ≤ 4. All *Cutibacterium* strains had an MIC of 25 μg/ml and slightly different MBCs (Table 1, Supplementary Data 1).

**Table 1:** MIC and MBC of prodigiosin against skin microbes and *Cutibacterium* species.

Strain	MIC (μg/mL)	MBC (μg/mL)	Ratio (MBC/MIC)	Antimicrobial activity
*Acinetobacter johnsonii* KCTC 12405^T^	3.125	25	8	Bacteriostatic
*Corynebacterium striatum* ATCC 6940	6.25	12.5	2	Bactericidal
*Cutibacterium acnes* KCTC 3314	25	50	2	Bactericidal
*Cutibacterium acnes* KCTC 3320	25	25	1	Bactericidal
*Cutibacterium avidum* KCTC 5339	25	50	2	Bactericidal
*Cutibacterium granulosum* KCTC 5747	25	100	4	Bactericidal
*Micrococcus luteus* KCTC 3063^T^	12.5	50	4	Bactericidal
*Staphylococcus epidermidis* KCTC 1917	12.5	50	4	Bactericidal
*Streptococcus mitis* KCTC 13047^T^	12.5	12.5	1	Bactericidal

### Growth of prodigiosin-treated *C. acnes*

**Figure 2:**
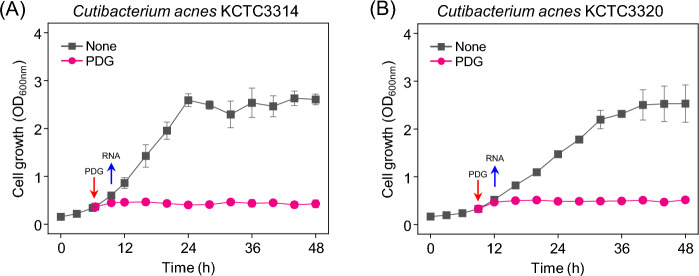
Cell growth of *Cutibacterium acnes* strains treated with prodigiosin (50 μg/mL) in BHI media. When OD_600nm_ was 0.4, prodigiosin was added, and RNA was extracted after 2 h.

Prodigiosin was added to the medium to confirm the effect on cell growth. At the early exponential phase (OD_600nm_ = 0.4) of the *C. acnes* strains (KCTC 3314 and KCTC 3320), the medium was divided, and one vial was added with 50 μg/ml prodigiosin solution. The other vial was added with ethanol to obtain a final concentration of 4%. Without prodigiosin, *C. acnes* KCTC 3314 and KCTC 3320 strains exhibited a stationary phase after the exponential growth (specific growth rates [μ] = 0.144, 0.137, respectively). However, when prodigiosin was added, the OD hardly increased (Fig. 2, Supplementary Data 2). Therefore, the growth of *C. acnes* was severely inhibited by prodigiosin.

### Transcriptomic analysis of prodigiosin-treated *C. acnes*

**Figure 3:**
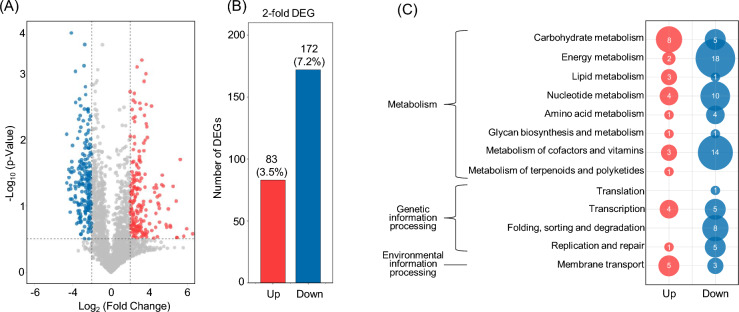
Overview of gene expression analysis. (**A**) The volcano diagrams of differential expressed genes (DEGs) in *Cutibacterium* after prodigiosin treatment. The horizontal axis shows the log2 value taken from the fold changes of gene expression; the vertical axis presents the statistically significant degree of changes in gene expression levels. The points represent genes. Gray dots indicate no significant expression genes, red dots refer to upregulated differential expression genes, and blue dots correspond to downregulated differential expression genes. (**B**) The number of twofold DEG genes. (**C**) Functional categories of twofold DEGs obtained from KEGG pathway analysis.

The genomes of *C. acnes* KCTC3314 and KCTC3320 strains exhibited a high degree of similarity, with an ANI value of 97.15%. To examine the specific alterations in gene expression induced by prodigiosin treatment, the transcriptomes of these two strains were compared. The global gene expression profiles after prodigiosin treatment were analyzed through RNA-seq. RNA-seq results were analyzed using the genome information of the KPA171202 strain (NCBI Accession No. NC006085) whose genome information was published^19^. The expression profiles of 2,391 genes excluding pseudogenes (69) among a total of 2,460 annotated genes were analyzed. The genes that displayed an expression fold-change of > 2 were considered significant results, which accounted for 35% of the total transcriptome. A total of 255 DEGs (10.7% of the total genes) were identified by the adjusted ∣log_2_(fold change)∣ > 1 and *Ρ* < 0.05 (Fig. 3A); among them, 83 and 172 genes were upregulated and downregulated, respectively (Fig. 3B, Supplementary Data 3).

### Functional classification of DEGs based on COG category and KEGG pathway analysis

A total of 255 DEGs were subjected to the COG protein category annotation and KEGG pathway analysis to further understand the function of the DEGs underlying the effect of prodigiosin on *C. acnes*.

In the COG category, 199 (78%) of 255 DEGs were annotated (Supplementary Fig. S3). The number of downregulated genes was more than that of upregulated ones in most categories, especially in categories such as “energy production and conversion,” “lipid transport and metabolism,” and “translation, ribosomal structure, and biogenesis” (Supplementary Fig. S3).

A total of 255 DEGs were mapped to the KEGG database and then examined via KEGG pathway enrichment analysis to understand the involved pathways. Of the 255 DEGs, 108 genes (42.4%) were mapped (Supplementary Data 4). Slightly more genes were upregulated in “carbohydrate metabolism,” but more genes were downregulated in most metabolism pathways. “Energy metabolism,” “nucleotide metabolism,” and “metabolism of cofactors and vitamins” were particularly more downregulated pathways (Fig. 3C).

Through COG analysis, more gene products were functionally classified, but one gene product belonged to several categories. Therefore, we analyzed the changes in gene expression through KEGG pathway analysis.

### DEGs involved in “carbohydrate metabolism”

**Figure 4:**
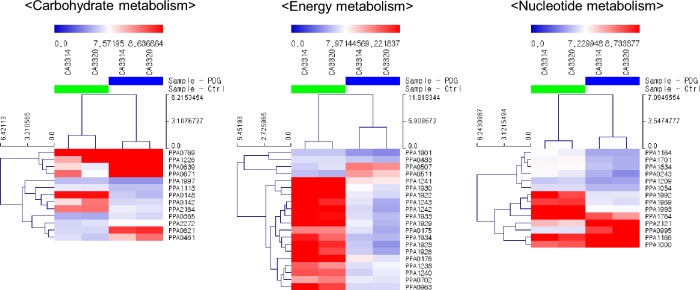
Heatmap of DEGs in control and prodigiosin treated cells in selected metabolisms.

In carbohydrate metabolism, eight genes were upregulated, and five genes were downregulated. The genes corresponding to amino sugar and nucleotide sugar metabolism (PPA0148, PPA1997, and PPA2164) were downregulated (Fig. 4, Supplementary Data 4). PPA0148, PPA1997, and PPA2164 encode UDP-*N*-acetylglucosamine 2-epimerase, *N*-acetylmannosamine-6-phosphate 2-epimerase, and β-*N*-acetylglucosaminidase, respectively.

### DEGs involved in “energy metabolism” and “nucleotide metabolism”

In the “energy metabolism” pathway, the genes encoding oxidative phosphorylation had related components. For example, (1) ATP synthase (PPA1238, 1240-1243), (2) cytochrome oxidase (PPA0175, PPA0176, and PPA0702), and (3) NADH dehydrogenase complex (PPA1901, PPA1922, PPA1926, PPA1928-1930, PPA1933, and PPA1934) were significantly downregulated (Fig. 4). The expression levels of nitrogen metabolism-related genes PPA0507 and PPA0511, which encode the nitrate reductase gamma subunit and nitrate/nitrite transporter, respectively, slightly increased.

“Nucleotide metabolism” also showed that most genes were downregulated. In purine metabolism, two genes were upregulated, and seven genes were downregulated. In pyrimidine metabolism, two genes were upregulated, and three genes were downregulated (Fig. 4).

### DEGs involved in “metabolism of cofactors and vitamins”

Most of the genes related to “metabolism of cofactor and vitamins” were also downregulated. Nicotinate and nicotinamide metabolism-related genes (PPA1318 and PPA0625) were downregulated, and they were involved in the synthesis of NADH complex, which is consistent with the downregulation of the overall energy metabolism. However, the expression of thiamine metabolism-related genes (PPA0885, PPA0110) increased. The genes involved in porphyrin metabolism were also highly downregulated (Supplementary Data 4). However, the expression of *deoR* (PPA0299), a transcriptional repressor of the porphyrin gene cluster, increased by about 4.6-fold, but the significance (*Ρ* = 0.5) was low^20^.

### DEGs involved in “genetic information processing” and “environmental information processing”

“Genetic information processing” includes categories such as transcription, translation, folding/sorting/degradation, and replication/repair. Since the growth of *C. acnes* was severely inhibited by prodigiosin, transcription and translation were generally downregulated. Although ribosomal proteins were significantly downregulated, the synthesis of aminoacyl-tRNA involved in translation slightly increased. The expression of translocase components related to protein export decreased. During homologous recombination in prokaryotes, the expression of RuvA (PPA1159) and RuvC (PPA1158), which participate in Holliday junction formation, significantly decreased (Supplementary Data 4).

“Environmental information processing” includes the categories such as membrane transport and signal transduction. In *C. acnes* treated with prodigiosin, only the expression of the ATP transporter, among the membrane transporters, changed. In particular, the expression of genes corresponding to phosphotransferase (PTS) did not change, and only the ABC transporters were detected.

### Stress-responsive RNA polymerase sigma factor (SigB)

In the prodigiosin-treated cells, PPA1031, which showed a high amino acid sequence similarity to the RNA polymerase sigma factor SigB, was significantly upregulated (Supplementary Data 4). SigB is an alternative sigma factor that is a master regulator of general stress response in *Bacillus* strains^21,22^.

### RT-qPCR validation

**Figure 5:**
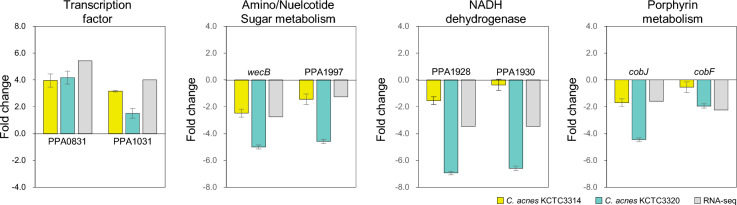
Validation of RNA sequencing data by quantitative RT-qPCR.

Several genes with different expression profiles were selected to validate the RNA-seq results. The transcription regulators PPA0831 (TetR family transcription regulator) and PPA1031 (RNA polymerase sigma factor SigB) were selected as upregulated genes. Although their expression levels differed between the two *C. acnes* strains, both genes were upregulated as shown in the RNA-seq results. The genes involved in (1) amino sugar and nucleotide sugar metabolism (*wecB*, PPA1997), (2) NADH dehydrogenase complexes (PPA1928, PPA1930), and (3) porphyrin metabolism (*cobJ*, *cobF*) were found. The transcription levels of the six selected downregulated genes were consistent with those of RNA-seq analysis (Fig. 5).

## Discussion

Antimicrobial activity of prodigiosin against various bacterial strains has been reported, and antibacterial mechanisms such as induction of autolysin in *Bacillus* species and formation of ROS in *Pseudomonas aeruginosa* have been suggested.^7,23^ We needed to test for skin commensal microbes for its potential cosmetic material. We confirmed that it has specific antibacterial activity to *Cutibacterium acne*s among skin commensal microbial strains, and determined MIC (~ 25 μg/mL) and MBC (~ 25–100 μg/mL) (Fig. 1, Table 1). While the most distinct and large inhibition zone was observed in *C. acnes* in the paper-disc assay, MIC was lower in other skin commensal microbes than in *C. acnes* possibly because the growth of the strain, the method of obtaining nutrients, and the degree of exposure to antibiotics are different in solid and liquid cultures. In addition, *C. acnes* strains undergo severe growth retardation by prodigiosin treatment (Fig. 2). We tried to understand the mechanisms of antibacterial activity by analyzing the global gene expression change caused by prodigiosin on *C. acnes*. *C. acnes* is a commensal bacterium found on the skin and has been studied as an opportunistic pathogen responsible for causing acne vulgaris. It is crucial to discover antibiotics and investigate their mechanism of action in order to effectively treat acne vulgaris caused by *C. acnes.*

*C. acnes* increases ribosome expression, oxidative phosphorylation, pyrimidine and purine metabolism, protein export/secretion, and glycolysis/glucose-producing genes in the exponential phase as growth phase-dependent transcription. It reflects active metabolism, replication, and protein translation^24^. When the *C. acnes* strain was treated with prodigiosin, its growth was severely inhibited, and growth phase-dependent expression, such as energy metabolism and nucleotide metabolism, was significantly reduced.

In addition, the expression of UDP-*N*-acetylglucosamine 2-epimerase (PPA0148), *N*-acetylmannosamine-6-phosphate 2-epimerase (PPA1997), and β-*N*-acetylglucosaminidase (PPA2164), which are enzymes involved in bacterial polysaccharide biosynthesis^25,26^, bacterial cell wall recycling, and flagellar assembly^27^ was decreased (Fig. 4). Prodigiosin depletes the lipopolysaccharide layer of *Escherichia coli* and *Bacillus cereus* because of its hydrophobic properties, affecting membrane integrity^8^. The downregulation of amino sugar and nucleotide sugar metabolism impairs the cell wall, prevents bacterial polysaccharide biosynthesis, and disrupts membrane integrity.

Porphyrin is a pro-inflammatory metabolite that plays roles in human disease manifesting inflammatory skin conditions. *Cutibacterium* strains that cause acne vulgaris produce high porphyrin levels; *Cutibacterium* strains with low porphyrin production are relatively abundant in healthy skin^28,29^. When prodigiosin was administered, the expression of porphyrin biosynthesis genes in the *C. acnes* strain was significantly downregulated. This result suggested that prodigiosin inhibited the production of pro-inflammatory metabolites such as porphyrin, thereby lowering the skin inflammatory response.

Among membrane transporters, the expression of cell division protein (PPA1352, PPA1353) decreased, which is considered to reflect cell growth retardation. The increased expression of the ABC transporters appears to be involved in antibiotic resistance mechanisms. ABC transporters can be utilized to acquire antibiotic resistance by mediating the release of antibiotics from bacterial cells by hydrolyzing ATP^30,31^.

Prodigiosin treatment of *Pseudomonas aeruginosa* reduces biofilm formation and affects cell proliferation by forming ROS^23^. Some genes corresponding to ROS-induced oxidative stress were identified in *C. acnes*^32^: PPA0097 (KatE; catalase), PPA1818 (superoxide dismutase), and PPA1939 (RoxP; a unique lineage-conserved antioxidant protein in *C. acnes*). RNA-seq revealed that the expression levels of these genes slightly increased, but they differed depending on the strains.

SigB, a stress response master regulator, increases susceptibility to heat, cold, acid, salt, and alcohol in bacterial strains, such as *Brevibacterium flavum* and *C. glutamicum*^33,34^. Therefore, the increased expression of SigB potentiated the stress response by prodigiosin.

Through RNA-seq analysis, we confirmed the characteristic changes in the expression of particular genes (i.e., a stress-responsive sigma factor gene, cell wall polysaccharide biosynthesis-associated genes, cellular energy metabolism-related genes, and porphyrin biosynthesis genes). Consequently, we demonstrated that prodigiosin is a highly effective antimicrobial agent against *Cutibacterium* sp. Our transcriptome data provided insights into the antibacterial mechanism and potential of prodigiosin as an anti-inflammatory substance against *Cutibacterium*.

## Materials and Methods

### Bacterial strains

The bacterial strains used in this study are shown in Table 2. The six strains identical to the six skin microorganisms constituting a commercially available skin microbiome whole cell mix (MAS-2005, ATCC, USA) were selected and purchased from the Korean Collection for Type Cultures (KCTC) at Korean Research Institute of Bioscience and Biotechnology (KRIBB; Jeongeup, Korea). *C. striatum* ATCC 6940 was purchased from American Type Culture Collection (ATCC; Manassas, VA 20108, USA). Additional *Cutibacterium* sp. was procured from KCTC for the antimicrobial assay; *Hahella chejuensis* was purchased from KCTC for prodigiosin production.

**Table 2:** Bacterial strains used in this study.

Strains	Source	Description
*Acinetobacter johnsonii* KCTC 12405^T^	KCTC	Skin microbiome whole cell mix (MAS-2005)
*Corynebacterium striatum* ATCC 6940	ATCC	Skin microbiome whole cell mix (MAS-2005)
*Cutibacterium acnes* KCTC 3320	KCTC	Skin microbiome whole cell mix (MAS-2005)
*Micrococcus luteus* KCTC 3063^T^	KCTC	Skin microbiome whole cell mix (MAS-2005)
*Staphylococcus epidermidis* KCTC 1917	KCTC	Skin microbiome whole cell mix (MAS-2005)
*Streptococcus mitis* KCTC 13047^T^	KCTC	Skin microbiome whole cell mix (MAS-2005)
*C. acnes* KCTC 3314	KCTC	*Cutibacterium* sp.
*Cutibacterium avidum* KCTC 5339	KCTC	*Cutibacterium* sp.
*Cutibacterium granulogum* KCTC 5747	KCTC	*Cutibacterium* sp.
*Hehella chejuensis* KCTC 2396	KCTC	Prodigiosin producer

### Culture conditions

*Cutibacterium* species were streaked and grown on reinforced clostridial medium (RCM) agar (Cat. No. 218081; BD Difco™, NJ, USA) at 37 °C under anaerobic conditions. A single colony of *C. acnes* was inoculated into a 125 mL butyl rubber-stoppered serum vial containing 25 mL of RCM broth, and the vial headspace was filled with N_2_ gas. The cells were grown anaerobically at 37 °C with shaking at 200 rpm for 48 h to produce a seed culture. Seed culture (1 mL) was used to inoculate a 125 mL butyl rubber-stoppered serum vial containing 100 mL of RCM or brain heart infusion (BHI; Cat. No. 237500; BD Difco™, NJ, USA) broth.

*H. chejuensis* KCTC 2696 cells were streaked and grown on marine agar (Cat. No. 2216; BD Difco™, NJ, USA) at 30 °C. Prodigiosin was extracted from the culture of KCTC 2696 cells^12^. For lab-scale flask cultures with 100 mL of marine broth and polyurethane (PU) foam cubes (Jeongan Sponge Co., Ltd., Korea; density, 25 kg/m^3^; size, ~ 1 cm^3^) were used to absorb prodigiosin. They were then incubated at 30 °C for 48 h with shaking at 200 rpm, and PU foam cubes were filtered through gauze to remove the culture medium. Prodigiosin was extracted from PU foam cubes by using a Soxhlet extractor with ethanol. Culture conditions and extraction methods for large-scale cultures using 200 L bioreactor were previously described^35^. The amounts and purity of prodigiosin extracts were determined by LC–MS analysis.

Four skin microbiome strains (*A. johnsoii*, *C. striatum*, *M. luteus*, and *S. epidermidis*) were streaked and grown on a TSA agar (Cat. No. 211825; BD Difco™, NJ, USA) at 37 °C under anaerobic conditions. *S. mitis* was streaked on the TSA agar wrapped for microaerobic culture at 37 °C. A single colony was inoculated into a 250 mL Erlenmeyer flask containing 25 mL of TSB broth. Cell growth was measured in terms of optical density at 600 nm by using a Libra S70 spectrophotometer (Biochrom, UK). The cell cultures were diluted at 1:10 by using the same media to measure the optical density accurately.

### Prodigiosin quantitation

Prodigiosin concentration was measured via high-performance liquid chromatography (Agilent 1100 Series HPLC System; Agilent, CA, USA) by using a C18 column (WAT05427, 100 Å, 5 μm, 4.6 × 250 mm; Waters Corp., MA, USA). Isocratic elution was performed at 25 °C with a flow rate of 0.8 ml/min by using a methanol:acetonitrile:distilled water (1:1:2, v/v) solution (pH adjusted to 3.6 with acetic acid) as a mobile phase.

### Disc diffusion assay

A disc diffusion assay was performed to evaluate the antibacterial activity. Filter-paper discs were wet with 100% ethanol (control) and prodigiosin solution and then dried. Each test cell suspension (1 mL; 10^6^ CFU/mL) and soft agar (14 mL; 0.7%) were mixed and poured on an agar plate (1.5%). After being hardened for 15–20 min, dried prodigiosin discs were applied to the inoculated agar. Inhibition zone diameters were measured in millimeters at 48 h.

### MIC and MBC tests

Minimal inhibitory concentration (MIC) and minimal bactericidal concentration (MBC) were analyzed using the dilution methods with modification. The prodigiosin was dissolved in 100% ethanol to prepare a stock solution. The 25 times concentrated prodigiosin stock solutions were diluted into various concentrations (0, 0.78, 1.56, 3.13, 6.25, 12.5, 25, 50, and 100 μg/mL) and added into appropriate broth in serum vials. Bacterial cells were inoculated into a final concentration of ~ 10^5^ CFU/mL and incubated at 37 °C for 48 h. Bacterial cell growth was measured using a Spectramax190 microplate reader (Molecular Device, CA, USA) at 600 nm (bacterial cells concentration) at the initial time point and after 48 h. Bacterial cell growth was corrected at OD_535nm_ of the medium control containing each prodigiosin concentration. The MIC endpoint was the lowest prodigiosin concentration at which no cell growth occurred in the tubes.

After the MIC of prodigiosin was determined, the culture broth from all the tubes that showed no visible bacterial growth was diluted, spread on appropriate agar plates, and incubated at 37 °C for 48 h. The MBC was defined as the lowest prodigiosin concentration that reduced the initial inoculum by ≥ 3 logs. A compound is considered bactericidal if the MBC/MIC ratio is ≤ 4. Bacteriostatic compounds have an MBC/MIC ratio of > 4^36^.

### RNA-seq

Culture condition for RNA isolation was described above. Specifically, when the OD_600nm_ was 0.4, 4 mL of prodigiosin stock solution (1.25 mg/mL, dissolved in 100% ethanol) was added to make the final concentration 50 μg/mL in medium. For the control (none), 4 mL (final 4%) of an ethanol solution without prodigiosin was added. After adding ethanol or prodigiosin solution and culturing for 3 h, the culture medium was centrifuged (12,000 rpm, 30 min, 4 °C) to obtain cell pellets. Cell pellets were frozen by liquid nitrogen and crushed using a mortar and pestle. Total RNA was isolated using Trizol reagent (Invitrogen, MA, USA), which contains a mixture of guanidine isothiocyanate, phenol, and chloroform. Subsequently, clear aqueous phase was collected and precipitated by ethanol. RNA quality was assessed using an Agilent 2100 bioanalyzer with RNA 6000 Nano Chip (Agilent Technologies, Amstelveen, The Netherlands), and RNA was quantified using a ND-2000 spectrophotometer (Thermo Inc., DE, USA).

RNA-seq was commercially commissioned to ebiogen (ebiogen, Korea). For control and test RNAs, rRNA was removed using a Ribo-Zero magnetic kit (Epicentre, Inc., USA) from each 5 μg of the total RNA. A library was constructed using a SMARTer Stranded RNA-Seq kit (Clontech Laboratories, Inc., CA, USA) in accordance with the manufacturer’s instructions. rRNA-depleted RNAs were used for cDNA synthesis and shearing in accordance with the manufacturer’s instruction. Indexing was performed using Illumina indices 1–12. The enrichment step was conducted via PCR. Subsequently, the libraries were checked using an Agilent 2100 bioanalyzer (DNA high-sensitivity kit) to evaluate the mean fragment size. Quantification was performed using a library quantification kit with a Step One real-time PCR system (Life Technologies, Inc., USA). High-throughput sequencing was performed as paired-end 100 sequencing via HiSeq 2500 (Illumina, Inc., USA). RNA-seq data were deposited in the NCBI BioProject under the accession number PRJNA867520 (SRR20999606, SRR20999573, SRR20999605, and SRR20999604; Supplementary Data 5).

Low quality reads and adapter sequences were filter out by BBDuk (version 35.74) in bbmap (“forcetrimleft = 11 k = 13 ktrim = r qtrim = t trimq = 20 minlength = 20”). RNA-Seq reads were mapped using Bowtie2 (version 2.4.4) software tool to obtain the alignment file. Sorted Bam files were converted into a Bed file, and RNA-seq read were counts by comparing with the reference genome annotation file using BEDtools (coverage in BEDtools with default option). Differentially expressed genes were determined on the basis of counts from unique and multiple alignments by using EdgeR (version 3.28.0) within R (R development Core Team, 2016) via Bioconductor (Gentleman et al., 2004). The alignment file was used to assemble transcripts. Quantile normalization method was used to compare between the samples. Gene classification was based on searches performed using DAVID (http://david.abcc.ncifcrf.gov/).

The whole-genome sequences of *C. acnes* KPA171202 (GenBank accession: NC_006085.1) was used as a reference for genome mapping. The ANI (Average Nucleotide Identity) values of KPA171202 as reference genome with KCTC3314 (= ATCC6919, GCF_008728435.1), KCTC3320 (= ATCC11828, GCA_000231215.1) were 99.06, 97.54%, respectively. RNA-seq read mapping were 86.63–97.00% (Supplementary data 6). Genes with an adjusted ∣log_2_(fold-change)∣ > 1 and *Ρ* < 0.05 were identified as DEGs (Supplementary Data 3). Clusters of Orthologous Groups (COGs) of DEGs were annotated by using the COG database (https://www.ncbi.nlm.nih.gov/research/cog) for COG categories, and the ATGC database for *C. acnes* COG information (https://ftp.ncbi.nlm.nih.gov/pub/kristensen/ATGC/atgc_home.html; Kristensen et al., 2017). KEGG (Kyoto Encyclopedia of Genes and Genomes) pathway analysis of DEGs was performed using the KEGG Mapper. A hierarchical clustering were performed using MeV 4.9.0 software.

### Real time-quantitative PCR (RT-qPCR)

The expression levels of the selected genes were measured via quantitative RT-PCR. Primers were designed using the IDT PrimerQuest™ Tool (https://sg.idtdna.com/PrimerQuest), and reactions were conducted on a CFX96 (Bio-Rad, Hercules, CA, USA) by using a RealHelix™ qRT-PCR kit (NanoHelix, Daejeon, Republic of Korea). RT-qPCR was conducted under the following conditions: cDNA synthesis (50 °C, 40 min), denaturation (95 °C, 12 min), and amplification for 40 cycles (95 °C, 20 s; 60 °C, 1 min). Raw fluorescence data were normalized against the 16S ribosomal RNA expression level. All primers used are shown in Supplementary Data 7.

### Supplementary Information

Supplementary Legends.

Supplementary Figures.

Supplementary Information.

## Data Availability

RNA-seq data were deposited in the NCBI BioProject under accession number PRJNA867520 (SRR20999606, SRR20999573, SRR20999605 and SRR20999604).
