# Supplementary material for: Transcriptomic analysis of the antimicrobial activity of prodigiosin against Cutibacterium acnes
Source: Sci Rep. 2023 Oct 13;13:17412. doi: 10.1038/s41598-023-44612-7 (PMC10576067; doi:10.1038/s41598-023-44612-7)
Supplement: Supplementary file 1 — Supplementary Legends. [file 41598_2023_44612_MOESM1_ESM.docx]

**Supplementary Figure legends**

**Supplementary Figure S1.** Paper-disc assay of the antibacterial activity of prodigiosin (0, 0.5, 1, 2, 5, and 10 μg) against *Cutibacterium* species.

**Supplementary Figure S2.** Cell growth of *Cutibacterium acnes* strains treated with prodigiosin (50 μg/mL) in RCM media. When OD_600nm_ was 0.4, prodigiosin was added.

**Supplementary Figure S3.** Functional categories of twofold DEG genes obtained from COG analysis.

**Supplementary Data list**

**Supplementary Data 1.** NCBI accession number of RNA-seq raw data.

**Supplementary Data 2.** Twofold DEGs (*Ρ* < 0.05) from RNA-seq analysis.

**Supplementary Data 3.** Primers used for RT-qPCR.

**Supplementary Data 4.** MIC and MBC tests of *Cutibacterium* species.

**Supplementary Data 5.** Cell growth and specific growth rate of *Cutibacterium* strains.

**Supplementary Data 6.** KEGG pathway analysis of twofold DEGs.
