# Supplementary material for: Transcriptomic analysis of the antimicrobial activity of prodigiosin against Cutibacterium acnes
Source: Sci Rep. 2023 Oct 13;13:17412. doi: 10.1038/s41598-023-44612-7 (PMC10576067; doi:10.1038/s41598-023-44612-7)
Supplement: Supplementary file 2 — Supplementary Figures. [file 41598_2023_44612_MOESM2_ESM.pdf]

Figure S1

Cutibacterium acnes
KCTC 3314

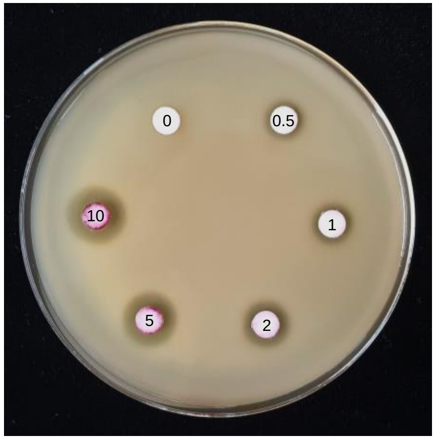

Cutibacterium acnes
KCTC 3320

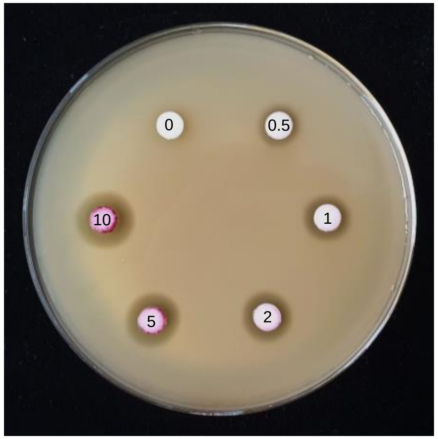

Cutibacterium avidum
KCTC 5339

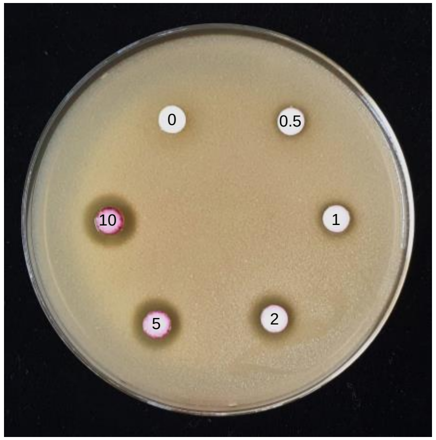

Cutibacterium granulosum
KCTC 5747

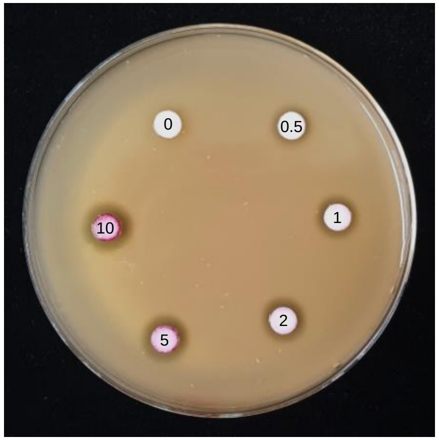

Figure S2

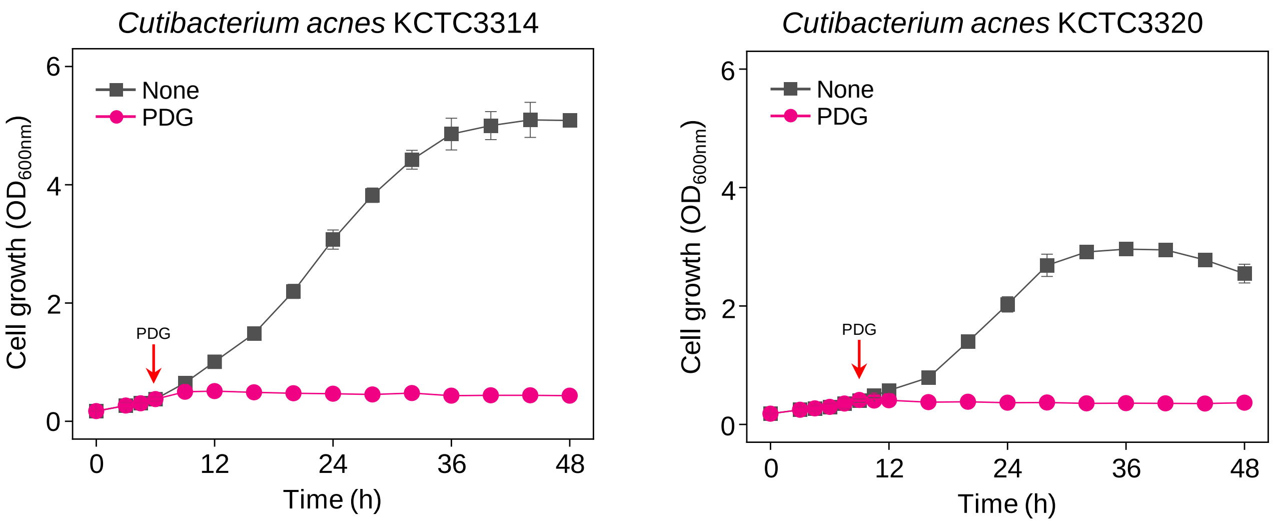

Cutibacterium acnes KCTC3314
Cell growth (OD600nm)
Time (h)
None
PDG
PDG
0
2
4
6
0
12
24
36
48
Cutibacterium acnes KCTC3320
Cell growth (OD600nm)
Time (h)
None
PDG
PDG
0
2
4
6
0
12
24
36
48

Figure S3

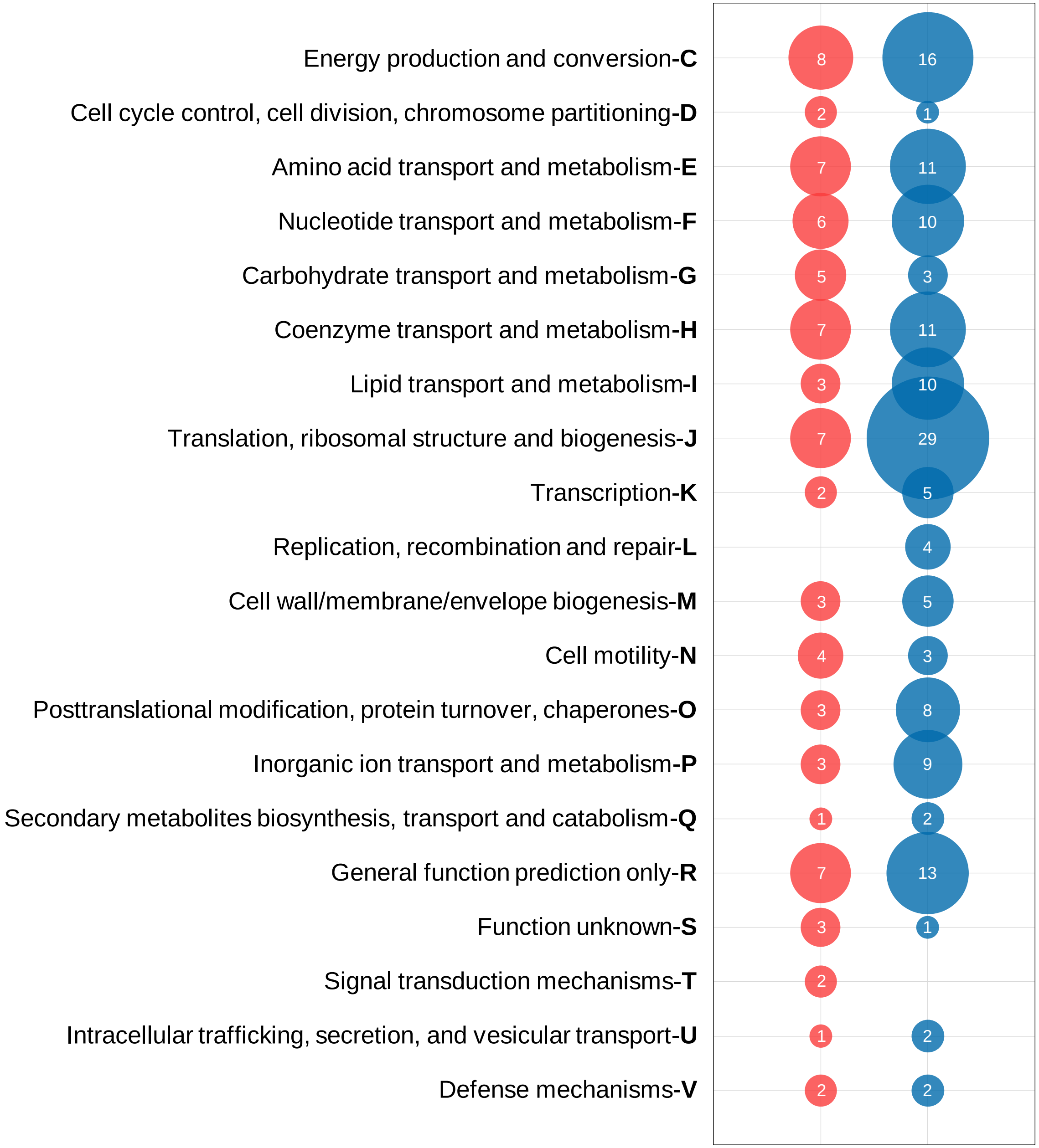

Energy production and conversion-**C**
8
16
Cell cycle control, cell division, chromosome partitioning-**D**
2
1
Amino acid transport and metabolism-**E**
7
11
Nucleotide transport and metabolism-**F**
6
10
Carbohydrate transport and metabolism-**G**
5
3
Coenzyme transport and metabolism-**H**
7
11
Lipid transport and metabolism-**I**
3
10
Translation, ribosomal structure and biogenesis-**J**
7
29
Transcription-**K**
2
5
Replication, recombination and repair-**L**
4
Cell wall/membrane/envelope biogenesis-**M**
3
5
Cell motility-**N**
4
3
Posttranslational modification, protein turnover, chaperones-**O**
3
8
Inorganic ion transport and metabolism-**P**
3
9
Secondary metabolites biosynthesis, transport and catabolism-**Q**
1
2
General function prediction only-**R**
7
13
Function unknown-**S**
3
1
Signal transduction mechanisms-**T**
2
Intracellular trafficking, secretion, and vesicular transport-**U**
1
2
Defense mechanisms-**V**
2
2
